# “Don Juan-Fracture” as a Hint to Aortic Isthmus Rupture

**DOI:** 10.1155/2014/758935

**Published:** 2014-11-18

**Authors:** Sirilak Suksompong, Benno von Bormann

**Affiliations:** Department of Anesthesiology, Siriraj Hospital, Faculty of Medicine, Mahidol University, 2 Prannok Road, Bangkoknoi, Bangkok 10700, Thailand

## Abstract

We report a case of thoracic aortic rupture after blunt trauma in a 23-year-old male patient. The initial investigation found no external injury or bleeding, only a slightly widened mediastinum and a broken left calcaneus. Abdominal lavage was negative, biochemistry was normal, and breathing and oxygenation were not compromised. When changing his position during diagnostics, the patient all of a sudden developed cardiac arrest and typical signs of hypovolemic shock. An immediate sternotomy was done without any further diagnostics on suspicion of aortic isthmus injury. A circular avulsion at the ligamentum arteriosum was found as assumed and repaired under cardiopulmonary bypass. The patient left the hospital for rehabilitation after 12 days in adequate health status. Biodynamics of blunt trauma after high-speed frontal impact and the relationship between calcaneus fracture, called “Don-Juan fracture,” and aortic rupture at the site of ligamentum arteriosum are discussed.

## 1. Introduction

The first report about traumatic injury of the thoracic aorta was made by Vesalius 1557 [1]. The natural history of the injury was eventually appreciated after the study of Parmley et al. in 1958 [2]. About 45% of blunt aortic injuries after car accident are caused by frontal impact [3]. “Don Juan” is a legendary, fictional libertine since 1630 (Lord Byron, Mozart, Moliere, and others). A calcaneus (Don Juan-) fracture is the fracture a lover could get by jumping down from the balcony of his mistress.

## 2. Case

A 23-year-old male patient was admitted late on a Sunday night into a University Hospital after car accident with frontal impact. He had been found by the rescue team sitting in the car with the seatbelt attached and the airbag inflated. On arrival, he was conscious but without memory of the accident. Initially, we found sinus tachycardia, severe hypertension (250/130 mmHg), a slightly widened mediastinum, and a broken left calcaneus, but nothing else of note (normal lab tests, negative abdominal lavage, and no drugs or alcohol). By instinct, we provided the patient with several IVs, an arterial and a central venous line, and a suprapubic bladder catheter which initially drained 220 mL of urine.

When lifting the patient from the gurney, he unpredictably developed severe shock with cardiac arrest. Cardiopulmonary resuscitation including endotracheal intubation was started immediately. Aortic bleeding was suggested as a possible cause, but none of the team present was sure as to whether a laparotomy or thoracotomy/sternotomy would be the most suitable approach. A coincidentally appearing senior cardiac surgeon considered the combination of slightly widened mediastinum and broken calcaneus to be indicative of thoracic aortic isthmus rupture, leading to the decision for immediate surgery. After sternotomy, cardiopulmonary bypass was established with cross clamping distal to the insertion of coronary arteries. Upon starting the heart-lung machine, a circular aortic defect, the size of about 1 cm in diameter, became apparent at the insertion site of the torn-off ligamentum arteriosum (former Ductus Botalli). Using a bent deBakey-Clamp, the defect was contemporaneously sealed and accessible. Surgery was uncomplicated with successful direct suture of the defect. After cessation of the bypass, the calcaneus fracture was treated with osteosynthesis. The patient was discharged for rehabilitation after fully recovering on day 12 after the operation.

## 3. Discussion

The aortic isthmus is affected in up to 80% of car accident victims with blunt aortic trauma [4]. Due to the anatomical condition with the ligamentum arteriosum attaching the aorta to the left pulmonary artery, heavy trauma of the thorax induces torsion and traction forces between the relatively mobile aortic arch and the distal descending aorta [5].

Severe nonpenetrating thoracic trauma and isthmus laceration are frequently accompanied by rib-, sternal-, and spine-fractures, lung injury, and ruptures of pericardial vessels, liver, spleen, and kidneys [1]. The only concomitant injury in our patient was a fracture of the left calcaneus, an injury that is not mentioned in context with aortic trauma within the entire literature. However, the calcaneus is one of the strongest bones of the body and it is short; therefore, an enormous impact is needed to fracture it. The patients' excessive high blood pressure after trauma was a secondary finding that had no impact on the diagnostic considerations. The phenomenon of “Hypertensive response to injury” was first described by a surgical research team during the war in Korea [6].

Considering the combination of calcaneus fracture and aortic rupture, we assume that the driver, realizing the inevitable collision, had his right leg bowed stepping with all his strength on the brake pedal, whereas the left leg was rigidly outstretched in a desperate effort to soften the impact. With the same intention, the arms were outstretched, the hands clinging round the steering wheel; the intactness of the arms has to be a result of the protective air bag function. In the moment of impact, when the body came to an abrupt halt the drivers' skeleton was rigidly stretched from head to heel. However, the inner organs, affected by strong vertical forces (responsible for the fracture of the calcaneus), moved on for a short but significant moment, following Newton's 1st law. The aorta being attached to the ligamentum arteriosum was exposed to an immense strain on the ligament leading to aortic rupture at the site of attachment [7, 8].

The patients' apparent stability at arrival was most likely due to an initially covered injury. Shorr et al. [9] reviewing 515 cases with blunt chest trauma and aortic injury reported that 339 patients had stable vital signs at arrival.

## 4. Conclusion

The patient reported owes his life to a physician capable of “lateral thinking.” An even slightly widened mediastinum after blunt thoracic trauma is generally a suspect for potential mediastinal bleeding and needs immediate clarification. In the rare case of an additional calcaneus fracture, particularly in young patients, therapists have to assume a high velocity impact that may lead to thoracic aortic rupture at the site of ligamentum arteriosum; the lack of obvious chest injuries, such as bruises and rib fractures, does not exclude aortic injury. Hemodynamics initially after blunt trauma is not reliable pointer to the severity of injury.
